# Use of diabetic healthcare according to the accessibility of diabetology services

**DOI:** 10.1093/eurpub/ckac131.309

**Published:** 2022-10-25

**Authors:** K Maláková, L Šídlo

**Affiliations:** Department of Demography and Geodemography, Charles University, Prague, Czechia

## Abstract

**Background:**

Diabetologists, as other specialists, are more likely concentrated in towns and cities rather than in the countryside so the people who live in these municipalities have a wider supply of health services. Our aim is to determine whether there are significant differences in the use of diabetology services between patients who live in the municipality with these services or not in Czechia.

**Methods:**

The sorted anonymized data obtained from the General Health Insurance Company of the Czech Republic (GHIC CR) were used. The studied patients were people with a diagnosis of type 2 diabetes mellitus (E11) who were insured by GHIC CR and used health services in 2019 in Czechia.

**Results:**

The distribution of providers of diabetology services (PDS) is relatively even throughout the country, and PDS are mainly concentrated in the municipality with a large population. In total, 52% of patients have the diabetologist in their municipality of residence. Patients living in the municipality with PDS have greater odds of using their services (OR 1.63, Cl 1.61-1.65). Specifically, 67% of the patients who have the diabetologist in their municipality of residence use diabetology services compared to 55% of the patients living in the municipality without PDS commute for diabetology services to the other municipality.

**Conclusions:**

The results show that diabetology services are concentrated mainly in towns and cities and patients living in the municipality with the diabetologist use more diabetology services compared to patients living in the municipality without them. At the same time, it seems that more than half of the patients in the municipality where is not PDS are willing and able to commute for diabetology services.

**Key messages:**

• Although patients living in the municipality without the diabetologist use diabetology services less often than with them, due to the commuting for healthcare, the differences are blurred.

